# An Evaluation of Higher Trainee Views on Clinical Posts in West, North and East Yorkshire Psychiatry Trainee Scheme

**DOI:** 10.1192/bjo.2022.289

**Published:** 2022-06-20

**Authors:** Christiana Elisha-Aboh, Laura Shaw, Rose Mozdiak, Sara Davies, Anilkumar Pillai

**Affiliations:** 1 Leeds and York Partnership NHS Foundation Trust, Leeds, UK, Leeds, United Kingdom; 2 Tees Esk & Wear Valley NHS Foundation Trust, UK, Knaresborough, United Kingdom; 3 General Adult Training Programme Director, Health Education England Yorkshire & Humber, Halifax, United Kingdom; 4 Old Age Training Programme Director, Health Education England Yorkshire & Humber, Bradford, United Kingdom

## Abstract

**Aims:**

Gathering honest feedback is challenging as trainees are often reluctant to do so due to the perceived impact on their reputation, future careers, and professional relationships. A lack of constructive feedback severely impacts future trainees and can prevent necessary improvements. There is considerable variation over collection of feedback. The aim of the project was to allow higher trainees and newly appointed consultants within two years of completing training, provide feedback on previous training posts in a confidential manner. The information obtained would be used to improve trainee experience, support a change in culture around feedback and highlight posts in need of input from Training Programme. Directors (TPDs).

**Methods:**

Anonymised questionnaires were sent to higher trainees and newly appointed consultants using a survey monkey link left open for a month. Reminders were sent via Medical Education, text messages, chats, and informal conversations. There were three basic open questions asked with free-text boxes. The questions were: What things were good about this post? What things could be improved? Would you recommend this post to a colleague? The data collected were in quantitative and qualitative formats.

**Results:**

We received 22 responses of 46 higher trainee posts within the scheme. The general themes from the project were that trainees wanted more focus on training rather than service provision, more independent working while still having good clinical support/supervision; based on their level of experience, better support to meet non-clinical Intended Learning Outcomes (ILOs) and ensuring a good balance of being busy while not finding it overwhelming. Trainees in community settings suggested allocation of selected cases focused on training experience, the opportunity to manage complex situations with supervision, being able to shadow and have joint reviews with consultants. The themes highlighted in the inpatient settings included having protected time to develop non-clinical ILOs, assuming greater leadership of clinical meetings, and having the opportunity to manage a patient from admission to discharge. A total of 4 posts were not recommended for reasons outlined above.

**Conclusion:**

Clearly there is a balance to be made between appropriate levels of independence and supervision. The vast majority of training posts reviewed have got the balance about right, however there are still some posts that require improvements. Careful consideration by both trainers and trainees needs to be given to various aspects of training, to achieve required ILOs, as not everyone fits the mould. This highlights the importance of creating individualised frameworks for trainee support and supervision.

